# Hidden Policy Attribute-Based Data Sharing with Direct Revocation and Keyword Search in Cloud Computing

**DOI:** 10.3390/s18072158

**Published:** 2018-07-04

**Authors:** Axin Wu, Dong Zheng, Yinghui Zhang, Menglei Yang

**Affiliations:** 1 National Engineering Laboratory for Wireless Security, Xi’an University of Posts and Telecommunications, Xi’an 710121, China; waxinsec@163.com (A.W.); zhengdong@xupt.edu.cn (D.Z.); kmelly@163.com (M.Y.); 2 Westone Cryptologic Research Center, Beijing 100070, China

**Keywords:** cloud computing, attribute-based encryption, direct revocation, keyword search, hidden policy

## Abstract

Attribute-based encryption can be used to realize fine-grained data sharing in open networks. However, in practical applications, we have to address further challenging issues, such as attribute revocation and data search. How do data users search for the data they need in massive amounts of data? When users leave the system, they lose the right to decrypt the shared data. In this case, how do we ensure that revoked users cannot decrypt shared data? In this paper, we successfully address these issues by proposing a hidden policy attribute-based data sharing scheme with direct revocation and keyword search. In the proposed scheme, the direct revocation of attributes does not need to update the private key of non-revoked users during revocation. In addition, a keyword search is realized in our scheme, and the search time is constant with the increase in attributes. In particular, the policy is hidden in our scheme, and hence, users’ privacy is protected. Our security and performance analyses show that the proposed scheme can tackle the security and efficiency concerns in cloud computing.

## 1. Introduction

With the application of intelligent terminals in our lives, a large amount of data can be generated quickly. These collected data are closely related to our lives. By analyzing personal data, one’s behavior can be predicted, and by analyzing the enterprise data, a lot of business secrets can be obtained which can pose a serious threat to individuals [1] or enterprises [2]. Furthermore, there are many threats to data privacy during the processes of data processing [3,4], data transmission [5], data storage [6,7], data search [8], data confidentiality [9,10] and data access [11,12]. Among these security problems, we focus on security issues in cloud storage and cloud computing.

While the rapid development of cloud computing brings convenience to enterprises and individuals because of its storage services, computing services, scalability and so on, data security and user privacy are also a big problem [13,14] owing to data being exposed in open network environments [15]. Encrypting data before uploading data to the cloud server can solve data security and user privacy issues very well [16,17]. However, the encryption of data causes the loss of some characteristics of plaintext, and data sharing among numerous data users becomes another problem. Fortunately, attribute-based encryption (ABE) [18] provides a good solution to the data sharing and access control on cloud storage and cloud computing. After Sahai et al. proposed the notion of ABE, much work was done to improve the function and efficiency of the ABE. For example, Li et al. [19] proposed a multi-authority, fine-grained access control scheme. Zhang et al. [20] proposed an anonymous access control scheme for proxy re-encryption. Shen et al. [21] proposed a data sharing scheme with anonymous tracking. These schemes are extensions of the ABE scheme and can be applied to some specific environments. In the scenario of data sharing, the Ciphertext Policy Attribute-Based Encryption (CP-ABE) [22] is more popular. In the CP-ABE system, the data owner specifies the access control structure related to ciphertexts. Only when the user attributes connected with the user secret key satisfy the access control structure, can the data be decrypted correctly. For example, Cai et al. [23] applied CP-ABE to the medical cloud which can help to improve the quality of medical services. Zhang et al. [24] applied CP-ABE to the mobile cloud computing, which makes it possible for resource-limited users to share data with others.

Although CP-ABE can bring a lot of convenience to our lives, there are still many problems to be considered in practical applications. For example, how can we ensure that revoked users cannot decrypt shared data? How do data users search for the data they want among massive amounts of data? In addition, in the CP-ABE system, the access control structure is also uploaded to the cloud server with the ciphertext, which may also leakage some sensitive information. In order to solve the above problems, the typical CP-ABE scheme is no longer suitable for the complex cloud computing environment. Therefore, searchable attribute-based encryption schemes (SABE) [25] and revocable attribute-based encryption schemes (RABE) [26] have been put forward.

In SABE [27,28], the data owner will upload the encrypted keyword index together with the ciphertext. When data users want to use data, he will generate a keyword trapdoor with his secret key, then uploads it to the cloud server. The cloud server checks whether the ciphertext containing the keyword index exists on the server without knowing the keyword. If it exists, the ciphertext will be returned to data users. Therefore, data users can retrieve the data they want based the keyword trapdoor. However, the search time in most searchable attribute-based encryption schemes increases with the number of attributes, which increases the burden on the server and reduces the user experience. In addition, when the access control structure is uploaded, it will also leakage some sensitive information.

The revocation scheme has practical application in dynamic networks and systems. For example, when a user leaves the system, the user identity is revoked in the system [29] which increases the security of the system. The RABE scheme can be divided into indirectly revocable attribute-based encryption (IRABE) schemes [30,31] and directly revocable attribute-based encryption (DRABE) schemes [32,33,34,35]. In the IRABE schemes, the revocation list is maintained by the authority center. When the user is removed from the system, the authority center updates the secret key of the non-revoked user. In DRABE schemes, the user’s revocation list is held by the user. When a user is revoked, the user’s private key does not need to be updated. Comparing the two schemes, the direct revocation scheme is more suitable for open network environments. In order to prevent the revoked users from decrypting the previous ciphertext, we can use the powerful computing power of cloud computing to update the ciphertext when the user is revoked from the system.

In order to make the data sharing scheme of CP-ABE more applicable to practical applications, it is necessary to propose a data sharing scheme with the functions of direct revocation and keyword search.

### 1.1. Our Contribution

In order to solve the problem described above and make the data sharing scheme of ABE more practical, we propose a hidden policy attribute-based data sharing scheme with direct revocation and keyword search (ABERS). Our scheme has the following advantages:

- Direct revocation of attributes: We use subset covering theorem to achieve the direct revocation of attributes. After revocation, there is no need to update the private key of the non-revoked user. In order to ensure that the users who have been revoked cannot decrypt the previous ciphertext, the ciphertext is updated.
- Fast keyword search: We use aggregation technology to achieve the fast search of keywords. Keyword search time is constant and will not increase with the numbers of attributes.
- Hidden policy: We use the AND gate access control structure to achieve the hidden policy. When the ciphertext is uploaded, the access control structure does not need to be uploaded. Thus, the function of the hidden policy can be realized.

### 1.2. Related Work

We review the work of the AND-gate attribute based encryption, the revocable attribute based encryption and the authorized keyword search in this section.

AND-gate attribute based encryption: Sahai and Waters [18] proposed the ABE scheme to solve data sharing and data access control. After Sahai et al. had proposed the ABE, much work was done to improve the function and efficiency of the ABE. In order to apply the function of the ABE scheme more flexibly, ABE was divided into key-policy ABE (KP-ABE) [36] and ciphertext-policy ABE (CP-ABE) [22,37]. In order to facilitate the application of terminal devices, some work was also done in references [38,39]. An AND-gate access structure ABE was introduced by Cheung and Newprot [40]. Unfortunately, there is no hidden access policy in this scheme. Due to the appearance of inner product encryption schemes, several other schemes [28,41,42] follow this structure, while hiding the access policy.

Revocable attribute based encryption: The RABE scheme is divided into IRABE schemes [30,31] and DRABE schemes [32,33,34]. In IRABE schemes, the revocation list is maintained by the authority center. When the user is removed from the system, the authority center updates the secret key of the non-revoked user. In DRABE schemes, the user’s revocation list is held by the user. When a user is revoked, the user’s private key does not need to be updated. In order to prevent revoked users from decrypting ciphertexts that existed before revocation, some work on the re-encryption proxy was done in reference [31] without interacting with data owners and in reference [43] without interacting with non-revoked users.

Authorized keyword search: The search encryption scheme can be traced back to Perrig et al. [44]. Unfortunately, the scheme has a high computational cost. To accelerate the search, Lee et al. [45] implemented search encryption through hash tables. To make the application scene more flexible, a keyword search encryption scheme based on a public key was proposed in reference [46,47]. In order to make the search more secure, the authentication search encryption scheme was proposed in reference [48,49]. Further work was done in references [50,51]. In reference [50], the authorized keyword search was implemented through ABE technology with multi-keywords. The scheme presented in reference [51] can be applied to multi-user and multi-owner scenes; however, it is not suitable for dynamic network environments.

## 2. Preliminary

In this section, we mainly introduce the basic knowledge about attribute revocation and keyword search.

### 2.1. Access Control Structure

The “AND gate” access structure [52] is described as follows: Let $S=(x_{1},x_{2},\ldots ,x_{n})$ represent an attribute list of a user. Let $W=(w_{1},w_{2},\ldots ,w_{n})$ represent an access policy. The attributes satisfy the access control structure, if and only if $x_{i}=w_{i},i\in \left[1,\ldots ,n\right]$. Because the access control structure and attribute set have the same structure, when uploading ciphertext, there is no need to upload the control structure. So, the hidden policy can be achieved.

### 2.2. Multilinear Maps

The concept of multilinear mapping was first proposed by Boneh and Silverberg, as the following [53]. First, run $\Gamma (1^{\lambda },n)$ to get G=(<$G_{1}$>,…,<$G_{n}$>), where λ is a security parameter. The description of the prime number group, $G_{i}$, whose order is $p>2^{\lambda }$ and contains the generator, $g_{i}$, of $G_{i}$, is expressed by $<G_{i}>$. A series of linear maps, $\left\{e_{i,j}:G_{i}\times G_{j}|i,j\geq 1;i+j\leq n\right\}$, are defined as follows:

$$e_{i,j}\left(g_{i}^{u},g_{j}^{v}\right)=g_{i+j}^{uv}\;\forall u,v\in Z_{p}.$$

We simplify the description as $e\left(g_{i}^{u},g_{j}^{v}\right)=g_{i+j}^{uv}$.

### 2.3. Subset Cover

**Figure 1:**
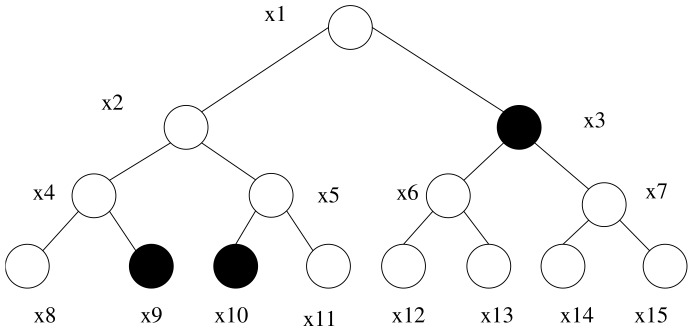
Subset cover.

First, we introduce the full binary tree *T* of depth *d*, in which two functions depth(x) and path(x) are involved. Both depth(x) and path(x) take node *x* as the input. The function depth(x) takes the depth of node *x* as the output. The function path(x) takes the path from the roo,t $P_{x,0}=root$, to the node, $P_{x,denpth(x)}=x$, as the output. The use of subset cover theorem to solve the user revocation was referred to by Naor et al. [54]. Let the leaf node express the user in the system. For a set of revoked users, *R*, we can get all paths, ${\left\{path\left(x\right)\right\}}_{\forall x\in R}$, of the revocation node x∈R. The cover(R) is the smallest set that can cover the unmarked nodes. For ease of understanding, we give a simple example, shown in Figure 1. Eight leaves $x_{8},\ldots ,x_{15}$ are contained in the full binary tree, *T*. If $R=\{x_{8},x_{11}\}$ is a revocation set. The paths of nodes $x_{8}$ and $x_{11}$ are $path\left(x_{8}\right)=\left(x_{1},x_{2},x_{4},x_{8}\right)$ and $path\left(x_{11}\right)=\left(x_{1},x_{2},x_{5},x_{11}\right)$, respectively. The cover(R) set is $\left\{x_{3},x_{9},x_{10}\right\}$. The non-revoked leaf nodes are covered by cover(R).

**Assumption 1.** *n-Multi-linearDecisionalDiffie–Hellman (n-MDDH): Run $\Gamma (1^{\gamma },n)$ to get G=(<$G_{1}$>,…,<$G_{n}$>). Select $v_{0},\ldots ,v_{n}\in Z_{p}$. Compute $g_{1}^{v_{0}},\ldots ,g_{1}^{v_{n}}$. For any poly-time algorithm, it is difficult with non-negligible advantage to tell $g_{n}^{\prod_{j\in [0,\ldots ,n]}v_{j}}$ from a random element in $G_{n}$. Please refer to [55] for more details.*

## 3. Definition

In this section, we mainly introduce the deployment of the model, the definition of the scheme and the security model of the scheme.

### 3.1. Deployment

**Figure 2:**
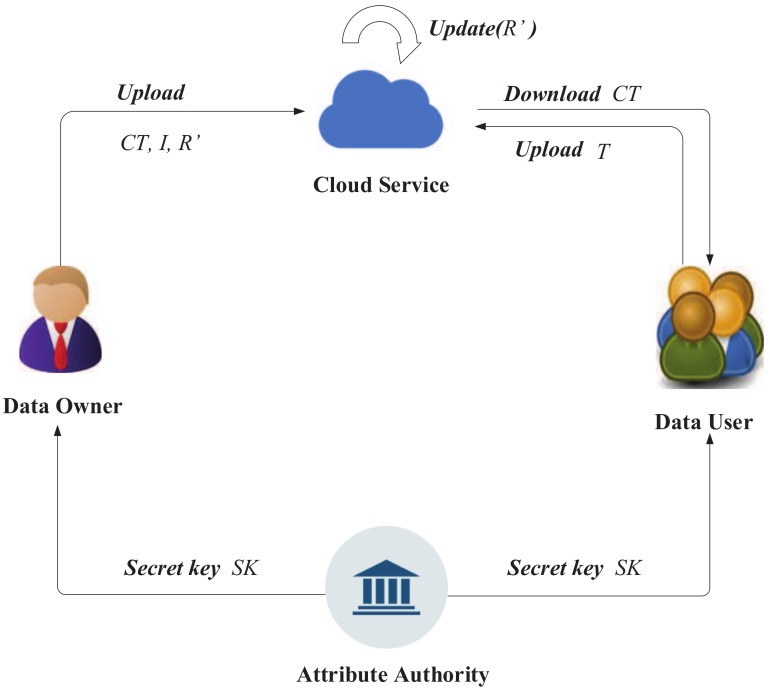
The data sharing system. *CT* is the ciphertext, *I* is the keyword index, *R′* is the revocation list, *T* is the keyword trapdoor and *SK* is the secret key.

ABERS can be applied to real environments. The data sharing system is shown in Figure 2. It involves four entities: data owner, data user, attribute authority and cloud server. Now, we will introduce their specific functions and functions.

- Data owner: The data owner is responsible for encrypting the data and generating the keyword index, *I*, and then uploading the ciphertext, CT, and keyword index, *I*. When the revocation list changes, the revocation list, $R{}^{\prime }$, is sent to the cloud server by the data owners.
- Data user: When data users want to download data, they should first use their own private keys to generate a keyword trapdoor, *T*, and then send *T* to the cloud server to check it. If the request is legal, then the desired data CT can be obtained.
- Attribute authority: The attribute authority is responsible for managing all users in the system, initializing the system, publishing the system’s public parameters, PK, and generating the secret key, SK, for the user.
- Cloud server: The cloud server is responsible for storing the ciphertext of the data owner. When the data user sends the keyword trapdoor to the cloud server, the cloud server searches for it. If the file exists, it is returned to the data user. When the new revocation list is received from the data owner, the cloud server updates the ciphertext with the $Updata(R{}^{\prime })$ algorithm.

### 3.2. Definition of the System Model

Our construction algorithm consists of the following eight algorithms.

$Setup(1^{\lambda },d,I,U)\to PK,MSK$: The algorithm takes the security parameters, λ, the depth of the tree, *d*, the set of the user identity, *I*, and the collection of attributes, *U*, as inputs with the common system parameters, PK, and the main secret key, MSK, as the outputs.

$Keygen\left(PK,MSK,S,id\right)\to SK_{S}$: The algorithm uses PK, MSK, the user attribute, *S*, and the user identity, id, as inputs, with $SK_{S}$ as the output.

$Encrypt\left(PK,M,W,R,w\right)\to CT_{W,R},I_{\omega }$: This algorithm uses PK, the message, *M*, an AND-gate access structure, *W*, a revocation list, *R* and the keyword, *w*, as inputs, with the ciphertext, $CT_{W,R}$, and keyword index, $I_{\omega }$, as the outputs.

$Trapdoor\left(SK_{S},w\right)\to t_{\omega }$: This algorithm takes the user’s secret key, $SK_{S}$, and a keyword, *w*, as inputs with a trapdoor, $t_{\omega }$, as the output.

$Test(I_{\omega },t_{\omega })\to 0$or 1: This algorithm takes the keyword index, $I_{\omega }$, and a trapdoor, $t_{\omega }$, as inputs with a Boolean value, {0,1}, as the output.

$Decryption(PK,CT_{W,R},SK_{S})\to m$ or ⊥: This algorithm takes $PP,CT_{W,R}$ and $SK_{S}$ as inputs, with *m* or ⊥ as the output.

$Update\left(CT_{W,R},R{}^{\prime }\right)\to CT_{W,R{}^{\prime }}$: This algorithm takes $CT_{W,R}$ and $R{}^{\prime }$ as inputs with $CT_{W,R{}^{\prime }}$ as the output.

### 3.3. Definition of System Security

The adversaries against the ABERS scheme include unauthorized data users and revoked data users. For unauthorized users, their attributes do not satisfy the access control structure. For revoked data users, their identities are in the revocation list. Both of them try their best to get the information of the ciphertext. Their behavior also includes a secret key recovery attack. They want to get a private key from a keyword trapdoor. The concrete models are as follows:

*Indistinguishability against chosen plaintext attack (IND-CPA):*

This security game is defined as follows:

- Init: The adversary, *A*, sends a revocation list, $R^{*}$, chosen by *A* to the challenger, *B*.
- Setup: *B* calls the algorithm $Setup(1^{\lambda },d,I,U)\to PP,MSK$, and then sends PP to *A*.
- Phase 1: The adversary, *A*, is able to ask *B* about the private key of user (S,id).
  When id∉R${}^{*}$, the enquiry is aborted. Otherwise, *B* calls the algorithm $Keygen\left(PP,MSK,S,id\right)\to SK_{S}$ and then sends $SK_{S}$ to *A*.
- Challenge: *A* sends two messages $m_{0}^{*}$, $m_{1}^{*}$ ($|m_{0}^{*}\left|=\right|m_{1}^{*}|$) and a challenge access structure, *W*, to *B*. *B* randomly selects b∈{0,1} and then calls the algorithm $Encrypt\left(PP,m_{b}^{*},W,R\right)\to CT_{W,R}$ and finally, sends $CT_{W,R}$ to *A*.
- Phase 2: *A* does the same inquiries as in Phase 1.
- Guess: *A* outputs the guess of *b* as $b{}^{\prime }\in \left\{0,1\right\}$.

In this game, the advantage of adversary *A* is defined as follows:

$$Pr_{A}=\left|Pr\left[b=b{}^{\prime }\right]-\frac{1}{2}\right|$$

**Definition** **1.** *If the advantage, $Pr_{A}$, of any polynomial-time adversary A is negligible, then the ABERS scheme is selectively indistinguishable under the (d+3)-MDDH assumption.*

*Indistinguishability against chosen keyword attack (IND-CKA):*

This security game is defined as follows:

- Setup: *B* calls the algorithm $Setup(1^{\lambda },d,I,U)\to PP,MSK$ and then sends PP to *A*.
- Phase 1: The adversary, *A*, is able to ask *B* about the private key of user (S,id). *B* calls the algorithm $Keygen\left(PP,MSK,S,id\right)\to SK_{S}$ and then sends $SK_{S}$ to *A*.
- Challenge: *A* sends two messages, $w_{0}^{*}$, $w_{1}^{*}$ ($|w_{0}^{*}\left|=\right|w_{1}^{*}|$), and a challenge access structure, *W*, to *B*. *B* randomly selects b∈{0,1} and then calls the algorithm $Encrypt\left(PP,M,W,R,w_{b}^{*}\right)\to CT_{W,R},I_{\omega }$ and finally, sends $I_{\omega }$ to *A*.
- Phase 2: *A* does the same inquiries as in Phase 1.
- Guess: *A* outputs the guess of *b* as $b{}^{\prime }\in \left\{0,1\right\}$.

In this game, the advantage of adversary *A* is defined as follows:

$$Pr_{A}=\left|Pr\left[b=b{}^{\prime }\right]-\frac{1}{2}\right|$$

**Definition** **2.** *If the advantage, $Pr_{A}$, of any polynomial-time adversary, A, is negligible, then the ABERS scheme is indistinguishable against the chosen keyword attack.*

*Selective security game on updated ciphertext:*

This security game is defined as follows:

- Setup: The adversary, *A*, sends two revocation lists, *R* and $R^{*}$, and an attribute, $S^{*}$, that chosen by *A* to the challenger, *B*. *B* calls the algorithm $Setup(1^{\lambda },d,I,U)\to PP,MSK$ and then sends PP to *A*.
- Phase 1: The adversary, *A*, is able to ask *B* about the private key of user $\left(S^{*},id\right)$. When $id\notin R^{*}$, the enquiry is aborted. Otherwise, *B* calls the algorithm $Keygen\left(PP,MSK,S,id\right)\to SK_{S}$ and then sends $SK_{S}$ to *A*.
- Challenge: *A* sends two messages, $m_{0}^{*}$, $m_{1}^{*}$ ($|m_{0}^{*}\left|=\right|m_{1}^{*}|$), and a challenge access structure, *W*, to *B*. *B* randomly selects b∈{0,1} and then calls the algorithm $Encrypt\left(PP,m_{b}^{*},W,R,w\right)\to CT_{W,R},I_{\omega }$ and $Update\left(CT_{W,R},R{}^{\prime }\right)\to CT_{W,R{}^{\prime }}$ and finally, sends $CT_{W,R{}^{\prime }}$ to *A*.
- Phase 2: *A* does the same inquiries as in Phase 1.
- Guess: *A* outputs the guess of *b* as $b{}^{\prime }\in \left\{0,1\right\}$.

In this game, the advantage of adversary *A* is defined as follows:

$$Pr_{A}=\left|Pr\left[b=b{}^{\prime }\right]-\frac{1}{2}\right|$$

**Definition** **3.** *If the advantage, $Pr_{A}$, of any polynomial-time adversary, A, is negligible, then the ABERS scheme has selective security under the (d+3)-MDDH assumption.*

## 4. Data Sharing System

In this section, we mainly introduce the concrete scheme, which contains the following seven algorithms *System initialization*, *User registration*, *Ciphertext upload*, *Trapdoor generation*, *Ciphertext retrieval*, *Ciphertext decryption* and *Ciphertext update*. The attribute authority executes the *System initialization* algorithm to generate public parameters and a master key for the system. Next, a secret key is generated by the attribute authority by running the *User registration* algorithm for each legitimate user based on their attributes. After that, ciphertext generated by the *Ciphertext upload* algorithm based on the access control structure can be uploaded to the cloud server to share data. If a data user wants to use data that is shared by a data owner, he first generates a keyword trapdoor with the *Trapdoor generation* algorithm based on his private key and keyword and uploads the keyword trapdoor to the server. After receiving the request, the cloud server checks whether the ciphertext containing the keyword trapdoor exists by calling the *Ciphertext retrieval* algorithm. If it exists, the ciphertext is returned to the data user. Then, the data user can decrypt the information with the *Ciphertext decryption* algorithm if his attributes satisfy the access control structure. In addition, when the cloud service receives the new revocation list from the data owner, the server updates the ciphertext with the *Ciphertext update* algorithm. The concrete implementation is as follows:

### 4.1. System Initialization

The attribute authority runs the Setup algorithm according to the system model definition. It runs the group generation algorithm to get $G=(<G_{1}>,\ldots ,<G_{d+3}>)$. Then, it selects a random number, $\alpha ,\beta ,a\in Z_{p}$ and a hash function, $H_{1}:{\left\{0,1\right\}}^{*}\to G_{1}$, $H_{2}:{\left\{0,1\right\}}^{*}\to G_{1}$. Finally, the PP and MSK are as follows:

$$PK=(G,g_{d+3}^{\alpha },g_{1}^{\beta },g_{1}^{a},H_{1},H_{2}).$$

MSK=(α,β).

### 4.2. User Registration

**Figure 3:**
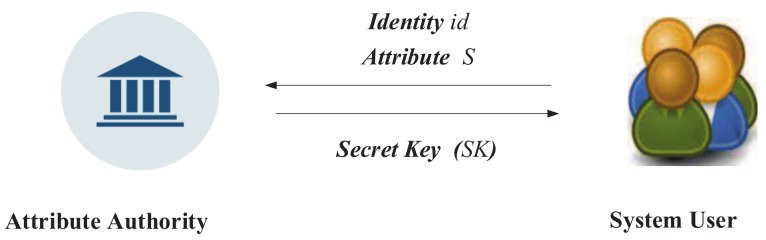
User registration.

At the user registration stage, the interaction between the attribute authority and the system user is as shown Figure 3—when the attribute authority receives the user’s attributes, *S*, and identity, id, the Keygen algorithm is called and returns the secret key, SK, to the system user safely.

The concrete algorithms are as follows: Suppose that the path of id is $path\left(id\right)=\left\{p_{id,0},\ldots ,p_{id,d}\right\}$, where $p_{id,0}=root$ and $p_{id,d}=x$. The algorithm sets $P_{id,-1}=g_{1}^{a}$. Then, it calls the following recursive algorithm: $P_{id,j}=e\left(H_{2}\left(P_{id,j}\right),P_{id,j-1}\right)$, for $j\in \left[0,d\right],P_{id,j}\in path\left(id\right)$. Then, for $\forall x_{i}\in S$, it randomly selects $r_{i}\in Z_{p}$. In addition, a random number, $r{}^{\prime }\in Z_{p}$, is selected. Finally, it calculates $r=\sum_{i=1}^{n}r_{i}$, $K_{0}=g_{d+2}^{\frac{\alpha +r}{\beta }}\cdot P_{id,d}^{r{}^{\prime }}$, $K_{1}=g_{1}^{r{}^{\prime }}$, $K_{2}=g_{1}^{\beta r{}^{\prime }}$, $k_{3}=\prod_{i=1}^{n}H_{1}{\left(x_{i}\right)}^{\beta }$, ${\left\{K_{i}=g_{d+2}^{r_{i}}\cdot H_{1}{\left(x_{i}\right)}^{r{}^{\prime }}\right\}}_{x_{i}\in S}$. The secret key is $SK_{S}=\left\{K_{0},K_{1},K_{2},K_{3},{\left\{K_{i}\right\}}_{x_{i}\in S}\right\}$.

### 4.3. Ciphertext Uploading

**Figure 4:**
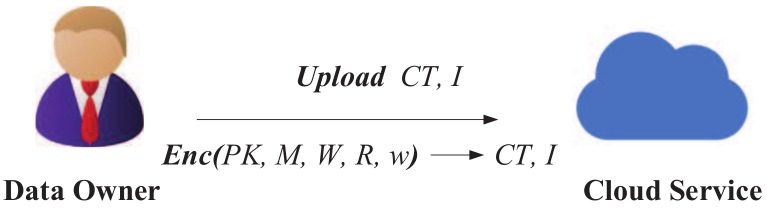
Ciphertext uploaded.

At the ciphertext uploading phase, the interaction between the cloud server and the data owner is as shown as Figure 4: The data owner calls the Encryption algorithm and then uploads the ciphertext, CT, and keyword index, *I*, to the cloud server.

The concrete algorithms are as follows: The algorithm randomly selects $s,s{}^{\prime }\leftarrow Z_{p}^{*}$ and then calculates $C_{0}=M\cdot g_{d+3}^{\alpha \cdot s}$, $C_{1}=g_{1}^{s}$, $C_{2}=g_{1}^{\beta s}$, $C_{1,i}=H_{1}{\left(W_{i}\right)}^{s}$, ${\tilde{C}}_{1}=e\left(g_{1}^{\beta },g_{1}^{ws{}^{\prime }}\right)$, ${\tilde{C}}_{2}=g_{1}^{ss{}^{\prime }}$.

Suppose the path of element x∈cover(R) is $path\left(x\right)=\left(p_{x,0},\ldots ,p_{x,depth(x)}\right)$, where $p_{x,0}$ represents root and $p_{x,depth(x)}=x$. Then, the algorithm sets $P_{x,-1}=g_{1}^{a}$. Finally, it calls the recursive algorithm $P_{x,j}=e\left(H\left(P_{x,j}\right),P_{x,j-1}\right)$ for j∈[0,d] and calculates $C_{2,i}=P_{x,depth(x)}^{s}$. The ciphertext and keyword index are as follows:

$$CT_{W,R}=\left(C_{0},C_{1},C_{2},\left\{C_{1,i},C_{2,i}\right\}\right).$$

$$I_{w}=\left({\tilde{C}}_{1},{\tilde{C}}_{2},\left\{C_{1,i}\right\}\right).$$

### 4.4. Trapdoor Generation

**Figure 5:**
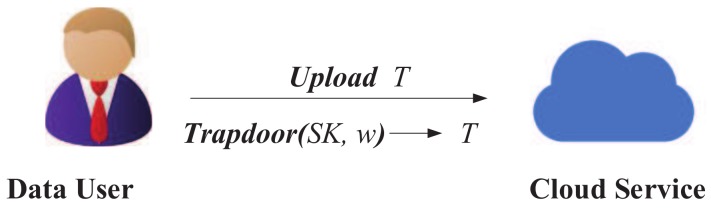
Trapdoor generation.

At the trapdoor generation phase, the interaction between the cloud server and the data user is as shown as Figure 5: The data user calls the algorithm Trapdoor, and then uploads the keyword trapdoor, *T*, to the cloud server.

The data user generates the keyword trapdoor with the following formula:

$$t_{w}=e\left(K_{3},g_{1}^{w}\right)=e\left(\prod_{i=1}^{n}H_{1}{\left(x_{i}\right)}^{\beta },g_{1}^{w}\right).$$

No information about *w* can be obtained from $t_{\omega }$.

### 4.5. Ciphertext Retrieval

The cloud server runs the Test algorithm according to the definition of the system model. It retrieves the file containing the keyword *w* with the following formula:

$$e\left({\tilde{C}}_{2},t_{w}\right)=e\left(\prod_{i=1}^{n}C_{1,i},\tilde{C}1\right).$$

When the equation is correct, it returns 1. The file exists on the cloud server. When the equation is wrong, it returns 0. The file does not exist on the cloud server.

The correctness of the phase *Ciphertext retrieval* is verified as follows:

$$\begin{array}{cc} e({\tilde{C}}_{2},t_{w}) & =e(g_{1}^{ss{}^{\prime }},e\left(\prod_{i=1}^{n}H_{1}{\left(x_{i}\right)}^{\beta },g_{1}^{w}\right))\\ & =e(\prod_{i=1}^{n}H_{1}{\left(x_{i}\right)}^{s},e\left(g_{1}^{s{}^{\prime }w},g_{1}^{\beta }\right))\\ & =e(\prod_{i=1}^{n}C_{1,i},{\tilde{C}}_{1}). \end{array}$$

### 4.6. Ciphertext Decryption

**Figure 6:**
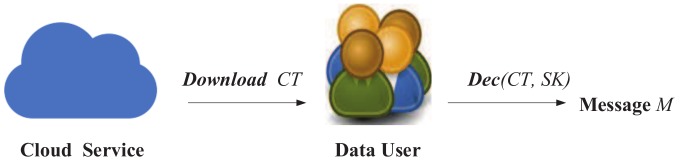
Ciphertext decryption.

At the ciphertext decryption stage, the interaction between the cloud server and the data user is as shown as Figure 6: The data user calls the Decrypt algorithm. If the user is legal, the ciphertext will be deciphered.

The concrete algorithms are as follows: If ∃i∈[1,…,n] $x_{i}\neq W_{i}$, the attribute list *S* does not satisfy the access control structure. The algorithm returns ⊥. When id∈R, The algorithm outputs ⊥. Otherwise, it calculates the following process.

If id does not belong to *R*, there will be a node, x∈(path(id)∩cover(R)), where $path\left(x\right)=\left(p_{x,0},\ldots ,p_{x,depth(x)}\right)$ and $path\left(id\right)=\left(p_{id,0},\ldots ,p_{id,d}\right)$. At the same time, there is $p_{id,j}=p_{x,j}$ for j∈[0,…,depth(x)].

The algorithm sets $P_{id,depth(x)}{}^{\prime }=P_{x,depth(x)}=C_{2,x}$. Then, it calls the recursive algorithm $P_{id,j}{}^{\prime }=e\left(H_{2}\left(P_{id,j}\right),P_{id,j-1}{}^{\prime }\right)$ for $j\in [depth\left(x_{i}\right)+1,\ldots ,d]$. The equation $P_{id,d}{}^{\prime }=P_{id,d}^{s}$ can be obtained.

Then, it calculates

$$\frac{e(K_{0},C_{2})}{\prod_{i=1}^{n}\frac{e(K_{i},C_{1})}{K_{1},C_{1,i}}\cdot e\left(P_{id,d}{}^{\prime },K_{2}\right)}=g_{d+3}^{\alpha s}.$$

Finally, the following formula is used to get the plaintext:

$$M=\frac{C_{0}}{g_{d+3}^{\alpha s}}.$$

The correctness of the *Ciphertext decryption* phase is verified as follows:

$$\begin{array}{cc} \prod_{i=1}^{n}\frac{e(K_{i},C_{1})}{e(K_{1},C_{1,i})} & =\prod_{i=1}^{n}\frac{e(g_{d+2}^{r_{i}}H_{1}\left(x_{i}\right)r{}^{\prime },g_{1}^{s})}{e{\left(g_{1}^{r{}^{\prime }},H_{1}\left(w_{i}\right)\right)}^{s}}\\ & =\prod_{i=1}^{n}e\left(g_{d+2}^{r_{i}},g_{1}^{s}\right)\\ & =g_{d+3}^{sr}. \end{array}$$

$$\begin{array}{cc} \frac{e(K_{0},C_{2})}{\prod_{i=1}^{n}\frac{e(K_{i},C_{1})}{e(K_{1},C_{1,i})}\cdot e\left(p_{id,d}{}^{\prime },K_{2}\right)} & =\frac{e(g_{d+2}^{\frac{\alpha +r}{\beta }}\cdot P_{id,d}^{r{}^{\prime }},g_{1}^{\beta s})}{g_{d+3}^{sr}\cdot e\left(p_{id,d}^{s},g_{1}^{\beta r{}^{\prime }}\right)}\\ & =g_{d+3}^{\alpha s}. \end{array}$$

### 4.7. Ciphertext Update

When the revocation is changed, the ciphertext stored on the cloud server will be updated. The cloud server runs the Update algorithm according to the definition of the system model. It inputs a ciphertext, $CT_{W,R}$, and a new revocation list, $R{}^{\prime }$, where $R\subset R{}^{\prime }$ outputs the updated ciphertext, $CT_{W,R{}^{\prime }}$.

If x∈Cover(R), x=y for $y\in Cover(R{}^{\prime })$. $C_{2,i}{}^{\prime }=C_{2,i}$ is set.

For x∈Cover(R), *y* is a child of *x*. Let $path\left(y\right)=path\left(x\right)⋃\left(p_{y,depth(x)+1},\ldots ,p_{y,depth(y)}\right)$ and set $p_{y,depth}{}^{\prime }=p_{x,depth}{}^{\prime }=C_{2,i}$. Then, it calls the recursive algorithm $P_{y,j}{}^{\prime }=e\left(H_{2}\left(P_{y,j}\right),P_{y,j-1}\right)$ for j∈[depth(x)+1,…,depth(y)]. Finally, it sets $C_{2,i}{}^{\prime }=P_{y,depth(y)}{}^{\prime }$, $C_{0}{}^{\prime }=C_{0}$, $C_{1}{}^{\prime }=C_{1}$, $C_{2}{}^{\prime }=C_{2}$, and $C_{1,i}{}^{\prime }=C_{1,i}$. The updated ciphertext is $CT_{W,R}=\left(C_{0}{}^{\prime },C_{1}{}^{\prime },C_{2}{}^{\prime },\left\{C_{1,i}{}^{\prime },C_{2,i}{}^{\prime }\right\}\right)$.

## 5. Security Proof

**Theorem** **1.** *The ABERS scheme is the IND-CPA security under (d+3)-MDDH assumption in the random oracle model.*

If the adversary, *A*, can break through our scheme with an advantage that we cannot ignore, a simulator, *B*, can call the Adversary, *A*, to break the (d+3)-MDDH assumption.

Simulator *B* inputs the group parameters, $\left(1^{\gamma },n\right)$, and instantiates the (d+3)-MDDH instance $\left(g_{1},g_{1}^{a_{0}},\ldots ,g_{2}^{a_{d+3}},Z\right)$. The game between the simulator *B* and the attacker *A* is as follows:

*Setup:* Adversary *A* selects a revocation list, $R^{*}$, and sends it to *B*. For each element, $id\in R^{*}$, in the revocation list, $R^{*}$, the simulator *B* sets $P_{R^{*}}={\left\{p_{id,i}\in path\left(id\right)\right\}}_{id\in R^{*},i\in \left[0,\ldots ,d\right]}$ and the hash functions $H_{1}$, $H_{2}$ are simulated as followed:

- $O_{H_{1}}$: When $H_{1}$ is called by the adversary, *A* (or *B*), a random number, $z_{i}\in Z_{p}$, is selected (unless it has already been done), and the simulator returns $g_{1}^{z_{i}}$ as a response to $H_{1}\left(x_{i}\right)$.
- $O_{H_{2}}$: When $p_{id,i}\in P_{R^{*}}$, $H_{2}$ is called by the adversary, *A* (or *B*), and a random number, $v_{id,i}\in Z_{p}$, will be selected (if it has already been done, the same result will be returned), and the simulator returns $g_{1}^{a_{i}+v_{id,i}}$ as a response to $H_{2}\left(p_{id,i}\right)$.
- When $p_{id,i}\notin P_{R^{*}}$, $H_{2}$ is called by the adversary, *A* (or *B*), a random number, $v_{id,i}\in Z_{p}$, will be selected (if it has already been done, the same result will be returned), and the simulator returns $g_{1}^{v_{id,i}}$ as a response to $H_{2}\left(p_{id,i}\right)$.

The challenger, *B*, randomly selects the random number, $\alpha ,\beta ,a\leftarrow Z_{p}$, and calculates $g_{d+3}^{\alpha },g_{1}^{\beta },g_{1}^{a}$ and then returns $\left(G,g_{d+3}^{\alpha },g_{1}^{\beta },g_{1}^{a},O_{H_{1}},O_{H_{2}}\right)$ to *A*.

Phase1&2: The adversary *A* makes the following enquiries to the challenger.

- When $id\notin R^{*}$, the enquiry is aborted.
- When $id\in R^{*}$, if *A* asks the challenger about the secret key of the user’s identity, id, and attributes, $S=(x_{1},x_{2},\ldots ,x_{n})$, random numbers, $r{{}^{\prime }}_{j}\in Z_{p}$ and $r_{i}^{j}\in Z_{p}\forall x_{i}\in S$, will be selected. Then, the simulator *B* calculates $r^{j}=\sum_{i=1}^{n}r_{i}^{j}$, $D=g_{d+2}^{\frac{\alpha +r^{j}}{\beta }}$, $K_{1}=g_{1}^{r_{j}{}^{\prime }},K_{2}=g_{1}^{\beta r_{j}{}^{\prime }}$, $K_{3}=\prod_{i=1}^{n}g_{1}^{z_{i}\beta }$ and $K_{i}=g_{1}^{r_{i}^{j}+z_{i}r_{j}{}^{\prime }}$.
- The path of id is represented as $path\left(id\right)=\left(p_{id,0},p_{id,d}\right)$ and then $H_{2}\left(p_{id,i}\right)=g_{1}^{\left(a_{i}+v_{id,i}\right)}$. After that, *B* computes $p_{id,d}=g_{d+2}^{a\prod_{i=0}^{d}\left(a_{i}+v_{id,i}\right)}$ by calling multi-linear maps on $g_{1}^{b},g_{1}^{a_{0}+v_{id,d}},\ldots ,g_{1}^{a_{d}+v_{id,d}}$ and $K_{0}=g_{d+2}^{\frac{\alpha +r^{j}}{\beta }}\cdot g_{d+2}^{b\prod_{i=0}^{d}\left(a_{i}+v_{id,i}\right)}$.
- Finally, the secret key, $\left\{K_{0},K_{1},K_{2},K_{3},{\left\{K_{i}\right\}}_{x_{i}\in S}\right\}$, is returned to *A*.

*Challenge:* The adversary *A* sends two messages, $m_{0}^{*}$, $m_{1}^{*}$ ($|m_{0}^{*}\left|=\right|m_{1}^{*}|$), and a challenge access structure, *W*, to *B*, *B* randomly selects b∈{0,1}, and the encryption process is as follows: $C_{0}=m_{b}^{*}\cdot Z$, $(C_{1}=g_{1}^{a_{d+3}}$, $C_{2}=g_{1}^{\beta a_{d+3}}$, $C_{1,i}=g_{1}^{t_{i}a_{d+3}}$. In addition, $P_{x,d}$ is generated according to the specified algorithm. $C_{2,i}=P_{x,d}^{a_{d+3}}$ is set. Finally, $\left(C_{0},C_{1},C_{2},\left\{C_{1,i},C_{2,i}\right\}\right)$ is sent to *A*.

*Guess:*$b{}^{\prime }\in \left\{0,1\right\}$ is output by *A*.

When $Z=g_{d+3}^{\prod_{j\in [0,\ldots ,d]}a_{j}}$, *A* plays the security game with *B*. When *Z* is a random number in a group, $G_{d+3}$, the information that $C_{0}$ contains $m_{b}^{*}$ is lost. Therefore, the simulator, *B*, can call the *A* to break the (d+3)-MDDH assumption. Because the assumption is difficult, our scheme is secure.

**Theorem** **2.** *Suppose q is a bound on the total number of group elements in the INK-CKA security game. The advantage in this security game is $O(q^{2}/p)$.*

Simulator *B* inputs the group parameters $\left(1^{\gamma },n\right)$ and instantiates the (d+3)-MDDH instance $\left(g_{1},g_{1}^{a_{0}},\ldots ,g_{2}^{a_{d+3}},Z\right)$. The game between the simulator *B* and the attacker *A* is as follows:

*Setup:* The hash function, $H_{1}$, is simulated as follows:

$O_{H_{1}}$: When $H_{1}$ is called by the adversary, *A* (or *B*), a random number, $z_{i}\in Z_{p}$, will be selected (unless it has already been done), and the simulator returns $g_{1}^{z_{i}}$ as a response to $H_{1}\left(x_{i}\right)$.

The challenger, *B*, randomly selects the random number, $\alpha ,\beta ,a\leftarrow Z_{p}$, and calculates $g_{d+3}^{\alpha },g_{1}^{\beta },g_{1}^{a}$ and then returns $\left(G,g_{d+3}^{\alpha },g_{1}^{\beta },g_{1}^{a},O_{H_{1}}\right)$ to *A*.

Phase1: The adversary, *A*, makes the following enquiries to the challenger.

The adversary *A* asks for the keyword, *w*, connected with $S=(x_{1},x_{2},\ldots ,x_{n})$ and the user’s identity, id, for *B*. The random numbers $r{{}^{\prime }}_{j}\in Z_{p}$ and $r_{i}^{j}\in Z_{p}\forall x_{i}\in S$ will be selected. Then, the simulator, *B*, calculates $r^{j}=\sum_{i=1}^{n}r_{i}^{j}$, $D=g_{d+2}^{\frac{\alpha +r^{j}}{\beta }}$, $K_{1}=g_{1}^{r_{j}{}^{\prime }},K_{2}=g_{1}^{\beta r_{j}{}^{\prime }}$, $K_{3}=\prod_{i=1}^{n}g_{1}^{z_{i}\beta }$ and $K_{i}=g_{1}^{r_{i}^{j}+z_{i}r_{j}{}^{\prime }}$.

Finally, the simulator *B* produces trapdoor $t_{w}$ as $t_{w}=e\left(K_{3},g_{1}^{w}\right)=e\left(g_{1}^{\beta \sum_{i=1}^{n}z_{i}},g_{1}^{w}\right)$. After that, the trapdoor $t_{w}$ is sent to *A*.

*Challenge:* The adversary, *A*, sends two keywords, $w_{0}^{*}$, $w_{1}^{*}$ ($|w_{0}^{*}\left|=\right|w_{1}^{*}|$) to *B*. At the same time, the challenge access control structure, *W*, will also be sent. *B* randomly selects $s,s{}^{\prime }\in Z_{p}$ and b∈{0,1}, and the encryption process is as follows: $C_{1,i}=g_{1}^{z_{i}s}$, ${\tilde{C}}_{1}=e\left(g_{1}^{\beta },g_{1}^{w_{b}^{*}s{}^{\prime }}\right)$, ${\tilde{C}}_{2}=g_{1}^{ss{}^{\prime }}$. The challenge index, $I_{w}^{*}$, is sent to *A*.

*Phase 2:* This stage is the same as *Phase 1*, but there is the restriction that the trapdoors of generated attributes that satisfy the access control policy have not been queried before.

*Guess:*$b{}^{\prime }\in \left\{0,1\right\}$ is output by *A*.

The Schwartz–Zipple lemma [56] points out that the probability of an “unexpected collision” occurring is, at most, $O(q^{2}/p)$.

**Theorem** **3.** *The ABERS scheme achieves selective security on updated ciphertext under the (d+3)-MDDH assumption in the random oracle model.*

We can see that any polynomial time adversary can not learn any information from the original ciphertext under Theorem 1. The key to proving Theorem 3 is determining whether the original ciphertext is distinguishable from the updated ciphertext.

Now let us take a look at whether the original ciphertext and the updated ciphertext generated by the same message, the attribute set, *S*, and the revocation list, $R{}^{\prime }$, are uniformly distributed.

The original ciphertext generated by calling $Encrypt(PP,M,W,R{}^{\prime },w)$ is

$$CT_{R{}^{\prime }}=\left(C_{0},C_{1},C_{2},\left\{C_{1,i},C_{2,i}\right\}\right),$$

where $C_{0}=M\cdot g_{d+3}^{\alpha \cdot s}$, $C_{1}=g_{1}^{s}$, $C_{2}=g_{1}^{\beta s}$, $C_{1,i}=H_{1}{\left(W_{i}\right)}^{s}$ and $C_{2,i}=P_{x,depth(x)}^{s}$.

The original ciphertext generated by calling Encrypt(PP,M,W,R,w) is

$$CT_{R}=\left(C_{0},C_{1},C_{2},\left\{C_{1,i},C_{2,i}\right\}\right),$$

where $C_{0}=M\cdot g_{d+3}^{\alpha \cdot s^{*}}$, $C_{1}=g_{1}^{s^{*}}$, $C_{2}=g_{1}^{\beta s^{}}$, $C_{1,i}=H_{1}{\left(W_{i}\right)}^{s^{*}}$ and $C_{2,i}=P_{x,depth(x)}^{s^{*}}$.

The updated ciphertext generated by calling $Update(CT_{R},R{}^{\prime })$ is

$$CT_{R^{{}^{\prime \prime }}}=\left(C_{0}{}^{\prime },C_{1}{}^{\prime },C_{2}{}^{\prime },\left\{C_{1,i}{}^{\prime },C_{2,i}{}^{\prime }\right\}\right),$$

where $C_{0}=M\cdot g_{d+3}^{\alpha \cdot s^{*}}$, $C_{1}=g_{1}^{s^{*}}$, $C_{2}=g_{1}^{\beta s^{}}$, $C_{1,i}=H_{1}{\left(W_{i}\right)}^{s^{*}}$ and $C_{2,i}=P_{x,depth(x)}^{s^{*}}$ for $\forall id\in (Cover\left(R\right)⋂Cover\left(R{}^{\prime }\right))$, $C_{2,i}{}^{\prime }=C_{2,i}=P_{x,depth(x)}^{s^{*}}$, and $\forall id\in (Cover\left(R{}^{\prime }\right)-Cover\left(R\right))$, $C_{2,i}{}^{\prime }=P_{x,depth(x)}^{s^{*}}$.

The original ciphertext and the updated ciphertext have the same terms, and each term is blinded by random numbers. Therefore, the original ciphertext and the updated ciphertext have the same distribution. At this point, similar to the analysis in [43], if the adversary, *A*, can break through our scheme, the simulator will be able to break the (d+3)-MDDH assumption.

## 6. Comparison

In this section, we compare our scheme with some related schemes. We have chosen several representative solutions related to the keyword search of ciphertext [42,52,57] and direct revocation [43,57,58]. The results of the comparison are shown in Table 1. Table 1 compares the functional differences between our schemes and related schemes from the perspective of keyword search, fast keyword search, direct revocation, hidden policy, communication overhead and storage overhead. Compared with other schemes, our scheme has better function. It is more accurate than the scheme [52]. The communication cost of the keyword trapdoor is the same, but the functioning of our scheme is greater. Compared with other schemes, the storage cost of our scheme is not very large.

**Figure 7:**
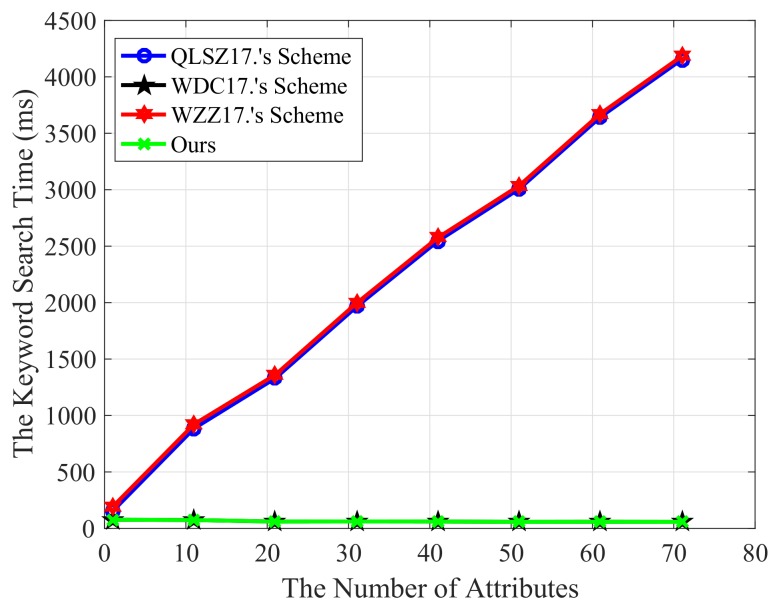
The comparison of keyword search performance.

Next, we compare the efficiency of the keyword search. In order to exclude other sources of interference and to make the result more accurate, we tested the schemes on the same platform, and the test results are shown in Figure 7. Figure 7 compares our scheme’s search efficiency with refs. [42,52,57]. We can see that, compared with schemes [42] and [57], the keyword search efficiency in our scheme is very high. The search time cost does not increase linearly with the number of attributes in ciphertext policies, which is not enabled in [42,57]. This is because our search scheme uses aggregated search key technology without pairing the secret key components with the corresponding ciphertext components. In the process of keyword trapdoor generation, only one linear pair operation is needed. In the process of ciphertext retrieval, by comparing whether the results of two pairs of linear pairs are equal, we can determine whether the required ciphertext exists. Although our scheme has the same efficiency in the search phase as that shown in reference [52], our scheme is more functional. From the point of view of function and efficiency, our scheme is more applicable to the practical environment.

## 7. Conclusions and Future Work

In this article, we have put forward a hidden policy attribute-based data sharing scheme with direct revocation and keyword search. The scheme has the following advantages. First, it uses subset covering theorem to achieve the direct revocation of attributes. After revocation, there is no need to update the private key of a non-revoked user. In order to ensure that the users who have been revoked cannot decrypt the previous ciphertext, the ciphertext is updated. In this way, some secret keys do not match some ciphertext, and users who are revoked can not decrypt the previous ciphertext. In addition, when there is a user leaving the system, we just need to send the revocation list to the cloud server and let the cloud server update the ciphertext. Then, the private key of the non-revoked user does not need to be updated. Second, we use aggregation technology to achieve the fast search of keywords. In the process of keyword trapdoor generation, only one linear pair operation is needed. In the process of ciphertext retrieval, by comparing whether the results of two pairs of linear pairs are equal, we can determine whether the required ciphertext exists. So, the keyword search time is constant and does not increase with the number of attributes. Third, the AND gate access control structure is used to achieve the hidden policy. When ciphertext is uploaded, the access control structure does not need to be uploaded. Thus, the function of the hidden policy can be achieved. In brief, when a lot of data is being shared, our solution can provide a good solution.

When a user leaves the system, the user needs to interact with the cloud server, and then, the server updates the ciphertext. This not only increases the cost of communication and computing, but the revoked user can decrypt the former ciphertext before the ciphertext is updated which is a threat to the security of the system. If there is no need to update the ciphertext, the revoked user will not be able to decrypt the ciphertext at the moment of revocation. So, in future work, we will solve the problem of how to ensure that the user can not decrypt the previous ciphertext without updating the ciphertext.

## Figures and Tables

**Table 1:** Feature comparison of our scheme and other typical schemes ${}^{†}$.

Scheme	KS	FKS	DR	HP	CO	SO
[58]	×	×	√	×	−	(|L|+|C|+2)|G|
[43]	×	×	√	√	−	(|S|+|C|+2)|G|
[42]	√	×	×	√	(2|S|+|Z|+1)|G|	(|P|+|P||W|+2)|G|
[52]	√	√	×	√	|G|	(2|S|+5)|G|
[57]	√	√	√	×	(|N|+3|L|+|I|)|G|	(2|S|-2|R|+|W|+|M|+3)|G|
Our scheme	√	√	√	√	|G|	(2|S|+|C|+2)|G|

${}^{†}$ The symbol √ (resp. ×) represents the corresponding feature is (resp. is not) achieved in the scheme. KS means keyword search, FKS means fast keyword search, DR means direct revocation, HP means hidden policy, CO means communication overhead and SO means storage sverhead. |S| means the number of user attributes, |Z| means the bit length of an element of $Z_{p}$, |G| means the bit length of an element of $G_{i}$, |I| means the bit length of user ID, |L| means the number of rows of the access control matrix, |P| means the number of columns of the access control structure, |C| means the cardinality of cover(R), |R| means the cardinality of a revocation list, |M| means the maximum number of revoked users and |N| means the number of keywords.
